# Paradigm Shifts in Voluntary Force Control and Motor Unit Behaviors with the Manipulated Size of Visual Error Perception

**DOI:** 10.3389/fphys.2017.00140

**Published:** 2017-03-13

**Authors:** Yi-Ching Chen, Yen-Ting Lin, Gwo-Ching Chang, Ing-Shiou Hwang

**Affiliations:** ^1^School of Physical Therapy, Chung Shan Medical UniversityTaichung, Taiwan; ^2^Physical Therapy Room, Chung Shan Medical University HospitalTaichung, Taiwan; ^3^Physical Education Room, Asian UniversityTaichung, Taiwan; ^4^Department of Information Engineering, I-Shou UniversityKaohsiung, Taiwan; ^5^Institute of Allied Health Sciences, College of Medicine, National Cheng Kung UniversityTainan, Taiwan; ^6^Department of Physical Therapy, College of Medicine, National Cheng Kung UniversityTainan, Taiwan

**Keywords:** error, feedback, feedforward, force control, motor unit, electroencephalography

## Abstract

The detection of error information is an essential prerequisite of a feedback-based movement. This study investigated the differential behavior and neurophysiological mechanisms of a cyclic force-tracking task using error-reducing and error-enhancing feedback. The discharge patterns of a relatively large number of motor units (MUs) were assessed with custom-designed multi-channel surface electromyography following mathematical decomposition of the experimentally-measured signals. Force characteristics, force-discharge relation, and phase-locking cortical activities in the contralateral motor cortex to individual MUs were contrasted among the low (LSF), normal (NSF), and high scaling factor (HSF) conditions, in which the sizes of online execution errors were displayed with various amplification ratios. Along with a spectral shift of the force output toward a lower band, force output with a more phase-lead became less irregular, and tracking accuracy was worse in the LSF condition than in the HSF condition. The coherent discharge of high phasic (HP) MUs with the target signal was greater, and inter-spike intervals were larger, in the LSF condition than in the HSF condition. Force-tracking in the LSF condition manifested with stronger phase-locked EEG activity in the contralateral motor cortex to discharge of the (HP) MUs (LSF > NSF, HSF). The coherent discharge of the (HP) MUs during the cyclic force-tracking predominated the force-discharge relation, which increased inversely to the error scaling factor. In conclusion, the size of visualized error gates motor unit discharge, force-discharge relation, and the relative influences of the feedback and feedforward processes on force control. A smaller visualized error size favors voluntary force control using a feedforward process, in relation to a selective central modulation that enhance the coherent discharge of (HP) MUs.

## Introduction

Humans need to consciously monitor execution errors and interpret the information of the perceived errors (such as the size and direction) to adjust motor outputs during goal-directed movements. Modifying the motor plan with execution errors requires the integration of information from multiple senses. In a visuomotor task, the proprioceptive system can detect some intrinsic error information (Coombes et al., 2011), but target exactness is better defined with external visual feedback. When visual consequences are misaligned with visuomotor rotation, task performance is degraded by perceptual conflicts that augment perceived directional changes in motor errors (Mazzoni and Krakauer, 2006; Ogawa and Imamizu, 2013). Visual feedback must be dissociated from predicted sensory consequences to drive compensatory motor corrections (Roby-Brami and Burnod, 1995; Taylor et al., 2014). Another paradigm that can bias the visual consequences of a visuomotor task is manipulation of the size of error feedback, or virtually making the visual perception of errors different from what they really are. Although the virtual augmentation of the error size also causes perceptual conflicts, it does not degrade task performance, instead expediting perceptual learning of a new motor task with fewer execution errors (Emken and Reinkensmeyer, 2005; Patton et al., 2013; Abdollahi et al., 2014). Past research has shown that participants given error-enhancing feedback can more quickly adapt to a perturbation force pushing their hands away from a direction of motion during a point-to-point movement (Sharp et al., 2011; Patton et al., 2013). In support of increases in error-related medial frontal theta activity, it is presumed that error enhancement could inflate response conflicts and enhance attentional focus on the motor task (Gehring and Fencsik, 2001; Rodriguez-Fornells et al., 2002). Although behavior studies have implied that error-enhancing feedback could improve motor performance and have potential rehabilitative benefits (Wang et al., 2010; Israely and Carmeli, 2016), little evidence to date supports the restriction of size-dependent error correction to the physiological contexts of motor unit behaviors.

The planning of a given visuomotor task can be generalized to a shared model along a continuum from feedback to feedforward (Pew, 1974; Seidler et al., 2004). A paradigm shift in feedback and feedforward control depends on errors and environmental contexts. Error reduction through extended visuomotor practice associates with improvements in feedforward control and thereby reduces demand on the feedback process (Sakai et al., 1998; Hill, 2014). In contrast, a visuomotor task is predominated by the feedback process in the early practice trials, and the brain can tacitly use a sampled process to minimize the negative impacts from the delay in sensory feedback (Navas and Stark, 1968; Miall et al., 1993). Central to this interpretation are several studies that have emphasized the roles of force fluctuations as a corollary to fine-grained force-scaling (Slifkin et al., 2000; Vaillancourt et al., 2002; Chen et al., 2013). Under the visual feedback of appropriately high spatial resolution, complexity of force fluctuations increases that indexes error corrections with better exploiting visual cues (Lee Hong and Newell, 2008; Kuznetsov and Riley, 2010; Prodoehl and Vaillancourt, 2010). When feedforward control prevails, force fluctuations (or movement intermittency) become less evident during the pursuit of a predictable target (Miall et al., 1986; Sosnoff and Newell, 2005). Examinations of motor unit (MU) control have also revealed that the brain can mediate input excitation to motoneurons based on the perceived error and thereby maintain force at the target level via rate modulation and/or motor unit recruitment in various visual conditions (Kamen and Du, 1999; Contessa and De Luca, 2013). MUs discharge rhythmically during cyclic force-tracking (Iyer et al., 1994; Knight and Kamen, 2007; Erimaki et al., 2013). Fast cyclic force-tracking favors the use of predictive control, and MUs discharge more coherently at the target rate during faster force-tracking than during slower force-tracking (Sosnoff et al., 2005). In addition, spatial resolution could mediate the capacity for sensorimotor integration, resulting in distinctive motor unit discharges (Jordan et al., 2013) and frequency contents of common synaptic input to motoneurons (Laine et al., 2014) for a force task with visual feedback of high and low spatial resolutions.

This study aimed to investigate the neurophysiological mechanisms of error-enhancing and error-reducing feedback that underlie the rebalancing of the feedback and feedforward processes. In addition to the structural changes in force behaviors, this study characterized the variations in MU discharge, the force-discharge relationship, and the central modulation of MU discharges during cyclic force-tracking visually-guided by execution errors of different virtual sizes. It was hypothesized that (1) the phase-lag, size, and regularity of force output would vary with the size of error feedback, (2) the discharge variability and phase-locking activity of MUs to cortical oscillations in the contralateral motor cortex would vary with the size of error feedback, and (3) force-discharge relation would vary with the size of error feedback. Our findings on corrective force behaviors and motor unit physiology shed light on the respective roles of the feedback and feedforward processes in cyclic force-tracking using error-reducing and error-enhancing strategies.

## Methods

### Experiment procedure

The participants were 14 right-handed healthy adults (7 males and 7 females; mean age: 26.1 ± 1.3 years, range: 21–35 years old). The research project was approved by Institutional Review Board (IRB) at the National Cheng Kung University (NCKU) Hospital, Taiwan. All of the participants signed an informed consent form before the experiment, conforming to the Declaration of Helsinki.

The target task was cyclic isometric force-tracking with index finger abduction. This task required participants to couple force output with a 0.5 Hz sinusoidal signal in a range of 10 ± 1.25% maximal voluntary contraction (MVC). The small range of force fluctuations of 2.5% MVC confined force regulation to the modulation of the firing rate of motor units (MUs). The low exertion level of the cyclic force task, around 10% MVC, was intended to minimize overlapping of the motor unit potential and neuromuscular fatigue after a number of experimental trials. The target rate of 0.5 Hz favored the use of a combination of feedback and feedforward processes to control a force-tracking task (Pew, 1974). The participant was seated in a chair with his/her dominant forearm resting on a table, and the dominant hand was pronated with the palm and forearm firmly fixed within a thermoplastic splint. The index finger was held slightly abducted (5 degrees of abduction), and its abduction force was measured using a force transducer (Model: MB-100, Interface Inc., Scottsdale, AZ, USA). The force output was displayed on a 19″ video monitor located 50 cm from the participant's eyes. The vertical axis of the visual display was 23 cm and comprised 1,080 pixels.

**Figure 1:**
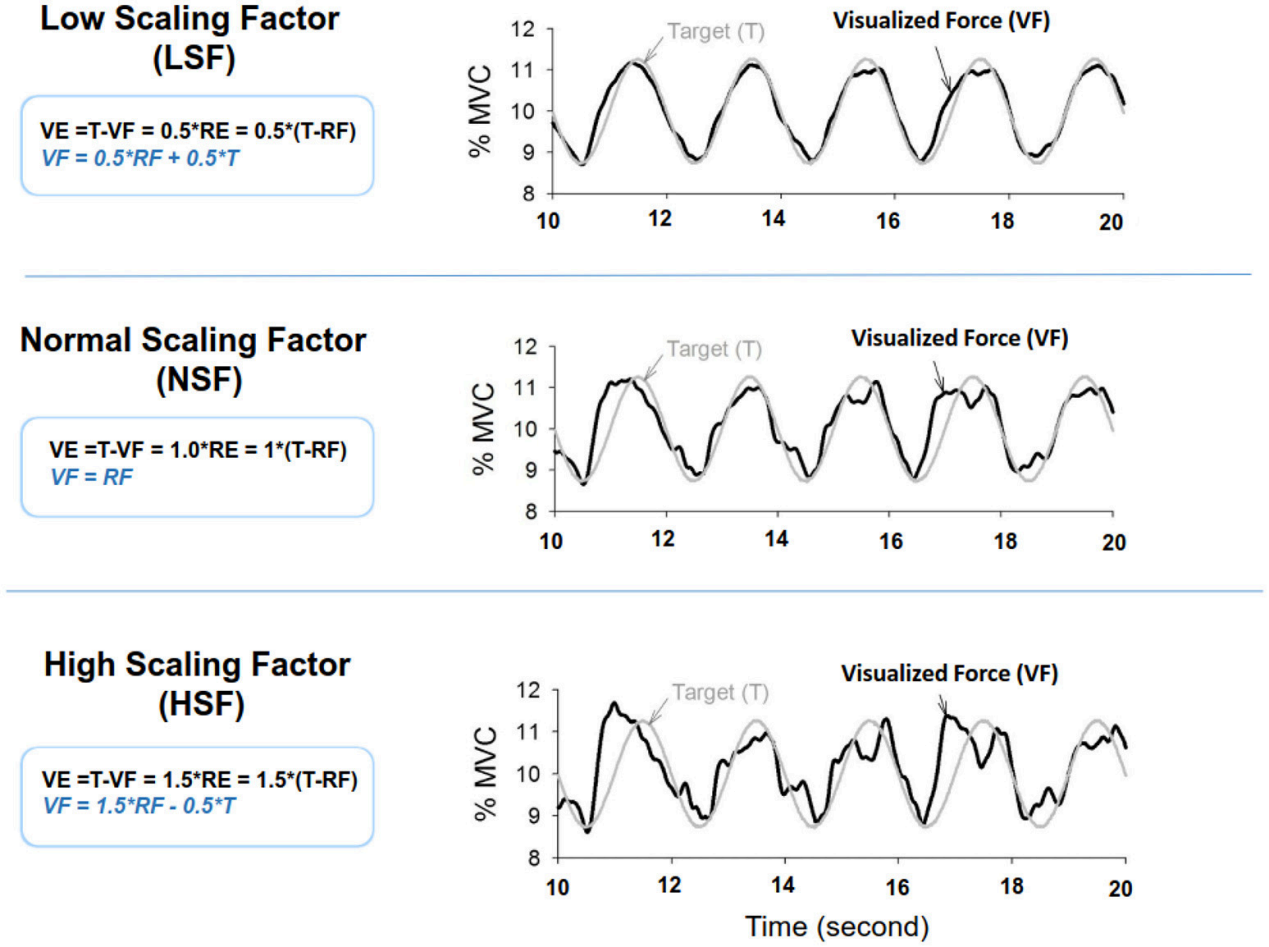
**Scaling factor alters the size of execution error during force-tracking with on-line visual feedback**. VE, visualized error; RF, real force; RE, real error.

**Figure 2:**
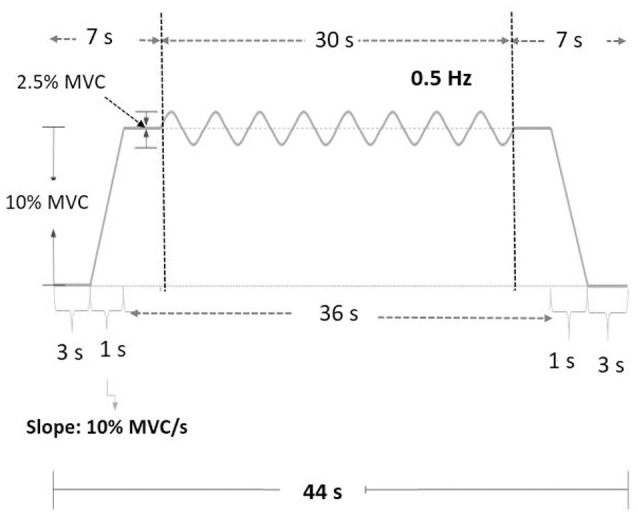
**Experimental protocol and the target signal of the force task**.

The MVC of the first dorsal interosseus (FDI) was predetermined as the peak force during three 3-s maximal contractions separated by 2 min pauses. The experiment commenced after a rest period of 20 minutes. There were three experimental conditions: low scaling factor (LSF), normal scaling factor (NSF), and high scaling factor (HSF) (Figure 1). The on-line execution error of the force task displayed on the monitor was virtually potentiated or condensed by scaling factors in each experimental condition. Before data collection, the participants were allowed three practice trials for each condition in a random order, during which they did not know that the visualized error feedback was manipulated. The size of the error feedback was displayed after real-time mathematical transformations of the force output (Hwang et al., 2017). In the LSF condition, the visualized force (VF) displayed on the monitor was equivalent to the sum of half the real force (RF) and half the target signal (T) (VF = 0.5^*^RF+0.5^*^T). Hence, the size of the visualized tracking error (VE) was half of the real error (RE) (VE = 0.5^*^RE). In the NSF condition, the visualized force was identical to the real force output, and the visualized error was equal to the real error (VE = RE). In the HSF condition, the VF was transformed with VF = 1.5^*^RF-0.5^*^T. The size of VE that the participant perceived was therefore augmented by 50% (VE = 1.5^*^RE). For all experimental conditions, the force output and target signal were displayed on a monitor, and the spatial resolution of the display of the target signal and force output was consistently set at approximately 50 pixels per 1% MVC. Hence, for all three feedback conditions, the vertical height between the maximum and minimum (2.5% MVC) of the target signal was consistently 125 pixels. The spatial resolution of the monitor used to display the target and force signals was identical across subjects for the three feedback conditions. The visual angle defined by the target signal was the same for all feedback conditions. Each experimental condition consisted of 5 contraction trials separated by 3-min pauses. The orders of the experimental trials were randomly assigned. The participants were instructed to produce isometric force by pushing their index fingers against the force transducer and to match the force produced to the target force line (Figure 2). After a latent period of 3 s, the participants started to increase the force output from zero to 10% MVC within 1 s. Following a short steady contraction of 3 s at 10% MVC, they varied the force output to match a 0.5 Hz sinusoidal target signal for another 30 s under visual guidance. Then they reduced the force output to the resting state within 1 s, and another 3-s latent period followed. One complete experimental trial was 44 s in length. The time window of interest was the 7–37th s (the period of load-varying isometric contraction around 10% MVC) of the experimental trial. The force output and target signal were sampled at 1 kHz by an analog-to-digital converter with 16-bit resolution (DAQCard-6024E; National Instruments Inc., Austin, TX, USA), controlled by a custom program running on a Labview platform (Labview v.8.5, National Instruments Inc., Austin, TX, USA).

### Electromyographic and electroencephalographic recordings

**Figure 3:**
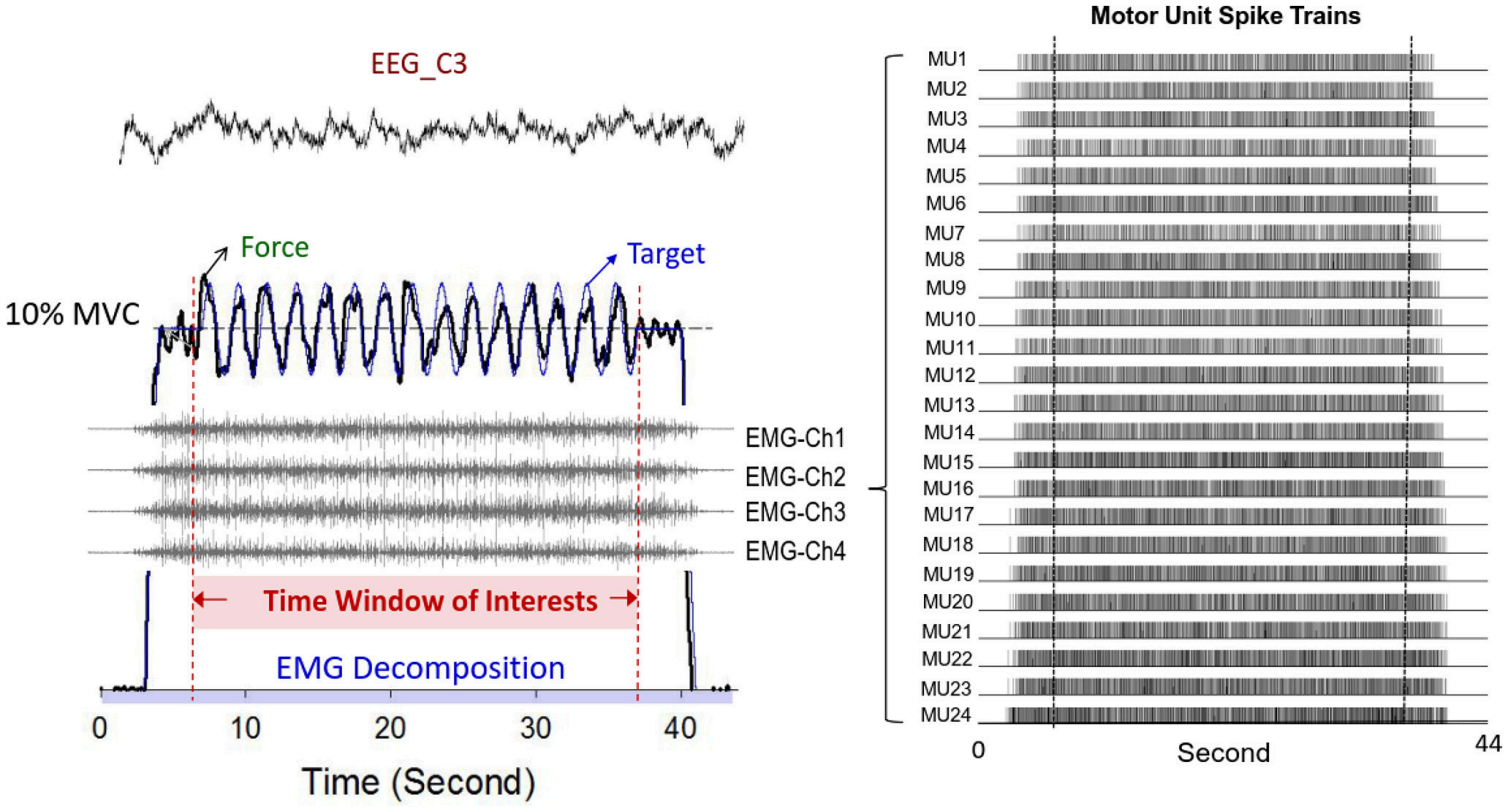
**Typical recording of physiological data**. Only force data, discharge variables, and EEG of the contralateral motor cortex in the time window of interest were presumably stable. The multi-channel surface EMG were decomposed to a number of motor unit spike trains with a state-of-the-art computational principle.

Synchronized with the force recording system, multi-electrode surface electromyography (Bagnoli sEMG system, Delsys Inc, Natick, MA, USA) was used to record the activity of the FDI muscle during dynamic force-tracking (Figure 3). Five surface pin-sensors with blunted ends (0.5 mm diameter) were placed at the corners and at the center of a 5 × 5 mm square. This special design was used to record the overlapping motor unit action potentials (MUAPs) of a relatively large number of MUs. The analog EMG signals from each pin-sensor were amplified (gain = 1,000) and band-pass filtered (cut-off frequencies 20 Hz and 450 Hz, 80 dB/octave roll-off) (De Luca et al., 2015b). By skin preparation and proper sensor application, baseline noises were strictly controlled to a peak-to-peak value of <20 μV. The conditioned EMG signal of each channel was sampled at 20 KHz to avoid phase skew across channels (De Luca et al., 2006; Nawab et al., 2010). Spatial filtering was administered by pair-wise subtractions of the voltages at the five pin-detections for the subsequent computational approach to decompose the single MUAP (De Luca et al., 2006; Hu et al., 2013). The software used for the post-decomposition processing of the action potential morphology was EMG works v.4.1 (Delsys Inc, Natick, MA, USA), which separated overlapping action potentials at various points in the EMG signal by using an artificial intelligence framework (De Luca et al., 2006; Nawab et al., 2010). Despite some criticisms (Farina and Enoka, 2011), the most recent studies have repeatedly shown that the computation algorithm can produce convincing decomposition results that discriminate overlapping action potentials for static isometric contraction (Nawab et al., 2004; De Luca et al., 2015a) from cyclic isometric contraction (De Luca et al., 2015b) via independent verification methods (Hu et al., 2013). Having accuracy that is comparable to that of the two-source test (De Luca et al., 2015a), the Decomposition-Synthesis-Decomposition-Compare (DSDC) test was used to validate the accuracy of the EMG decomposition of each motor unit action potential train (MUAPT) (De Luca et al., 1982, 2006). The entire data collection period (44 s) was identified with decomposition processing. This processing resulted in binary spike trains that coded the activations of all MUs with values of 0 or 1 (Figure 3). For each participant, the individual decomposition accuracies of all identifiable MUs from five experimental trials were averaged to represent the mean decomposition accuracy of all the experimental conditions. Previous studies have reported that the decomposition accuracy of MUAPTs using the same algorithm ranges from 92.5 to 97.6% (De Luca et al., 1982, 2006).

For EEG data acquisition, a pair of active Ag-AgCl electrodes (3 mm diameter; Model F-E9M-40-5, Grass, USA) were placed 1 cm apart on the C3 area, which was over the hand area of the primary motor cortex. The reference electrodes for the EEG were placed on the bilateral earlobes. After amplification of the recorded signal (gain = 5,000), the EEG signal was hardware-filtered in the frequency range of 0.01–100 Hz and 60 Hz (Model P511, Grass, USA). Off-line artifact-free EEG recordings were obtained using independent component analysis without prior knowledge of the contaminants (Urigüen and Garcia-Zapirain, 2015). Synchronized with the EMG system and force data, the EEG signal was sampled at 1,000 Hz.

### Force characteristics and motor unit discharges

The force signal was conditioned with a low-pass filter (cut-off frequency: 6 Hz) (Manjarrez et al., 2002; Lin et al., 2014) to discard involuntary tremulous movements at higher frequency bands (such as 8–12 Hz physiological tremor) that were functionally independent of the force-gradation of visuo-motor processes (Slifkin et al., 2000; Vaillancourt et al., 2002). Then we considered the force data in the time window of interest (the 7–37th s) to estimate the task error and force characteristics during the cyclic force-tracking (Figure 3). The force-tracking error was defined as the root mean square (RMS) of the mismatch between the target signal and real force output. The real force output after removal of a linear trend was the force output for subsequent force variable analysis, using ‘detrend’ command in Matlab software (Mathworks Inc., USA). The size and complexity of the force output were, respectively, assessed with RMS value (F_RMS_) and sample entropy (F_SampEn_) after a down-sampling process at 100 Hz (Lee Hong and Newell, 2008; Chen et al., 2013). Sample entropy measures the logarithmic likelihood that runs of patterns that are close to each other for m observations (within tolerance r) will remain close on subsequent incremental comparisons (Richman and Moorman, 2000). The mathematical formula of sample entropy was $SampEn\left(m,r,N\right)=-\text{log}\left(\frac{\overset{N-m}{\underset{i=1}{\sum }}A_{i}}{\overset{N-m}{\underset{i=1}{\sum }}B_{i}}\right)$, where *r* = 15% of the standard deviation of the force channel, *m* is the length of the template *(m* = 3), and *N* is the number of data points in the time series. *A*_i_ is the number of matches of the *i*th template of length *m* + *1* data points, and *B*_i_ is the number of matches of the *i*th template of length *m* data points (Pethick et al., 2015). A lower value represents greater regularity of force characteristics. In the time domain, we also assessed the lag time of the force output relative to the target signal by calculating the timing of the cross-correlation maximum of the two time-series. A negative lag time indicated that the force output led the target signal, and a positive lag time indicated that the force output lagged behind the target signal. The peak value of the cross-correlation (XC_FTmax_) indexed the temporal similarity between the conditioned force output and target signal. The force spectral profile of the force output was estimated with a fast Fourier transform and the Welch method (Hanning window, window length: 10 s, overlapping time segment: 1/5 × window length) with a spectral resolution of 0.1 Hz. The mean frequency (MF) and normalized amplitude of peak frequency at 0.5 Hz (PF_0.5Hz_)(amplitude of peak frequency at 0.5 Hz divided by all spectral areas) were determined. The change in mean frequency indexed the spectral shift of the force fluctuation profile. PF_0.5Hz_ reflected the degree of spectral resemblance of the force output to the target signal. For each participant, force fluctuation variables of the five contraction trials were averaged for each feedback condition.

In line with the truncated force data, we determined the discharge variables in the time window of interest from the decomposed EMG data using a total length of 44 s. The smoothed discharge rates of each MU during the time window were obtained by a smoothening procedure with a 400 ms Hanning window to suppress tremulous discharge (Negro and Farina, 2012). The temporal similarities of the smoothed discharge rates and target signal of each MU were assessed using peak cross-correlation (XC_DTmax_). Because the coherent activity of the detrended discharge rate varied among MUs, the types of MUs could be classified according to the XC_DTmax_. In this study, high phasic MUs (HP MUs) were empirically defined as the MUs that had the highest 20% XC_DTmax_ among all MUs because discharge of the defined HP MUs was later shown to vary with the scaling factors. The remaining MUs were low phasic MUs (LP MUs). For the three feedback conditions, the mean values of the inter-spike intervals (mean ISI) of all the MUs, HP MUs, and LP MUs were averaged across trials. The relationships of force and discharge coupling for all, the HP, and the LP MUs were characterized with the peak value of cross-correlation of the force output and the cumulative spike trains of the MUs (all MUs, the HP MUs, and the LP MUs) following low-pass filtering with a 400 ms Hanning window (Farina and Negro, 2015; Farina et al., 2016). The peak cross-correlation was averaged across the experimental trials of each feedback condition.

To investigate the central modulation and timing relationships between the cortical activity of the contralateral motor cortex and a single MU discharge (or EEG-SMU phase coupling) (Laine et al., 2012), the time-frequency representation of the EEG signal of the C3 electrode, *W(t,F)*, was decomposed with complex Morlet wavelets, $W\left(t,f\right)=\frac{1}{\sqrt{\left|a\right|}}\int_{-\infty }^{\infty }s\left(t\right)\varphi^{*}\left(\frac{t-b}{a}\right)dt$ where $\varphi^{*}\left(\frac{t-b}{a}\right)$: the complex conjugate of a Morlet mother wavelet, whose shape is a sinusoid weighted by a Gaussian kernel; *a*: scale factor; *t*: time translation; and *s(t)*: signal of interest (EEG C3). The instantaneous wavelet power *p*(*t*) = |*W*(*t*)| and the instantaneous phase. θ(t)=Tan-1(Re(W(t))Im(W(t))) Hence, the times of a single HP MU discharge could be mapped to the instantaneous phase of the spectral components [delta (1–3 Hz), theta (4–7 Hz), alpha (8–12 Hz), and beta (13–35 Hz)] of the EEG of the contralateral primary motor cortex. In this study, the Rayleigh z (*RZ*) statistic was used to test the significance of EEG-SMU phase coupling. The Rayleigh Z (RZ) statistic was mathematically formulated as, $RZ(f)=N*{\left\lfloor \frac{1}{N}*\left|\sum_{k\;=\;1}^{N}\frac{W(k,f)}{\left|W(k,f)\right|}\right|\right\rfloor }^{2}$ where *N* is the number of action potentials used in the analysis and *k* is the time at which each action potential occurred. RZ values ≥ 3 exceed the 95% confidence level (Fisher, 1993). That is, a significant phase-locking phenomenon exists between single HP MU and EEG spectral components, and MUAPs were unlikely to occur randomly with the instantaneous phase of the EEG. Although an HP MU could be significantly phase-locked to more than one spectral band of the EEG, only the peak Rayleigh Z of an individual MU was analyzed because it represented the maximal strength of the EEG-SMU phase coupling for brevity. For each participant, we averaged the peak Rayleigh Z of all HP MUs in the three feedback conditions and determined the percentages of the spectral bands wherein the peak Rayleigh Z occurred for the five experimental trials in each feedback condition. Signal processing was completed using Matlab (Mathworks Inc., USA).

### Statistical analysis

The major interest of this study was to investigate the differences in the force characteristics and discharge behaviors of MUs for the LSF, NSF, and HSF conditions. Repeated measures one-way ANOVA was used to contrast the force variables (force-tracking error, F_RMS_, F_SampEn_, XC_FTmax_, lag time, MF, and PF_0.5Hz_) among the LSF, NSF, and HSF conditions. Repeated measures one-way ANOVA was also used to examine the differences in decomposition (mean number of decomposed MUs and mean decomposition accuracy) and discharge variables (XC_DTmax_, mean ISI, peak Rayleigh Z, and the percentage of the spectral range wherein the peak Rayleigh Z took place) among the three feedback conditions. Finally, the force-discharge relations, in terms of cross-correlation peak for all, HP, and LP MUs among the LSF, NSF, and HSF conditions, were also examined using the same statistical approach. The levels of significance for the ANOVA was 0.05. The significance of the *post-hoc* test for the error size effect was *p* = 0.0167 using the Bonferroni correction (Dunn, 1961). Statistical analyses were performed in Statistical Package for Social Sciences (SPSS) for Windows v. 19.0 (SPSS Inc., USA). Data reported in the texts, figures, and tables without specific notations are presented as mean ± standard error.

## Results

### Task error and force characteristics

**Figure 4:**
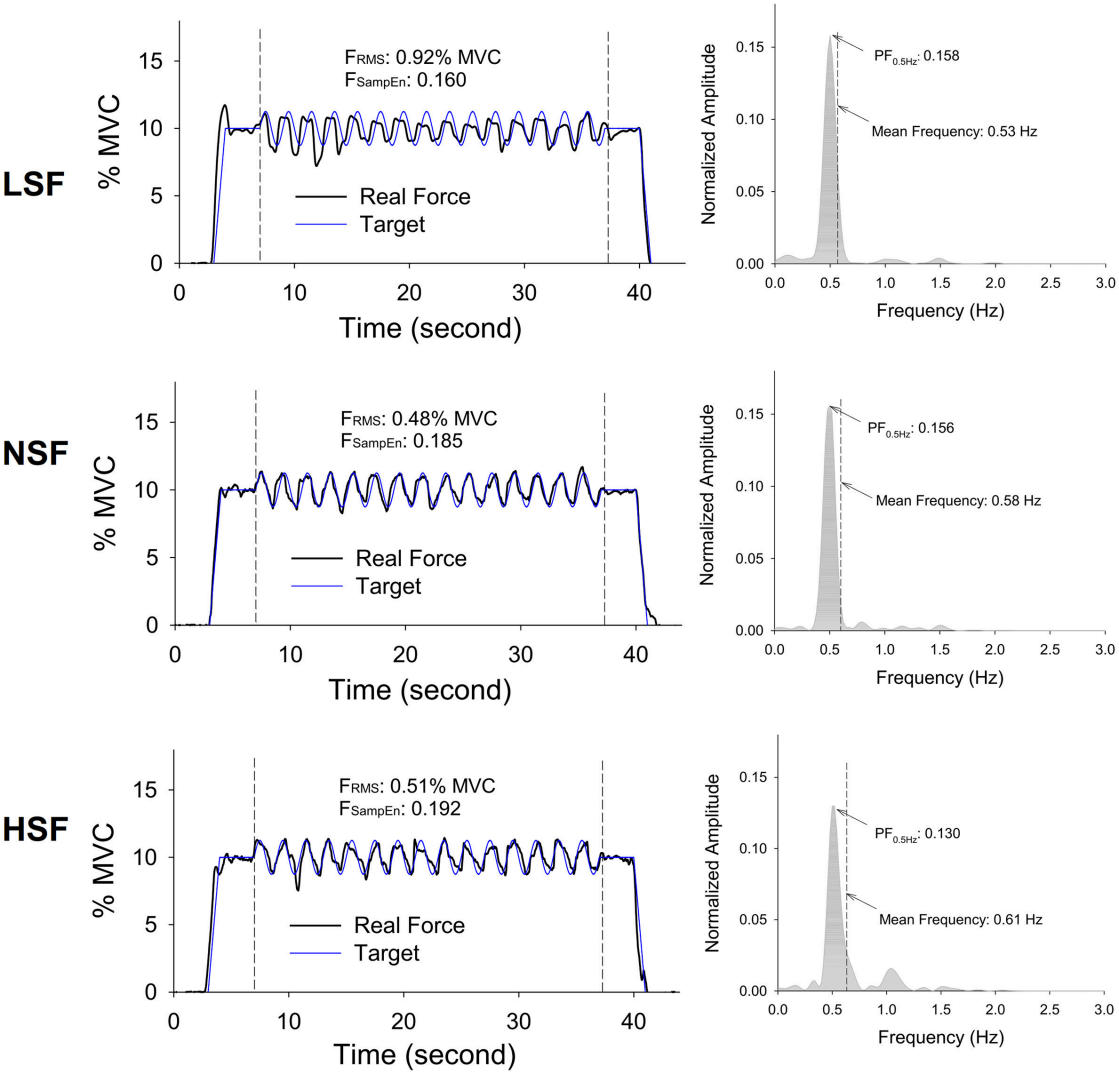
**Representative trials and pooled power spectra of force data of the times of interest from a typical participant for the low scaling factor (LSF), normal scaling factor (NSF), and high scaling factor (HSF) conditions**. The mean frequency of each pooled spectrum of force output is labeled. (F_RMS_: root mean square value of force output, F_SampEn_: Sample entropy of force output, PF_0.5Hz_: normalized amplitude of peak frequency at 0.5 Hz).

Figure 4 displays the force output and target signal of a representative trial and the pooled spectra of the force characteristics of five experimental trials from a typical participant in the LSF, NSF, and HSF conditions. The spectral profiles of the conditioned force outputs in the time windows of interest exhibited marked spectral peaks at the target rates with sub-band structures. Table 1 summarizes the results of the ANOVA test on variations in all the behavior variables with error scaling factor. The scaling factor significantly affected task performance and force structures in the time and spectral domains (*p* ≤ 0.001). In general, F_SampEn_, MF, and the lag time showed an increasing trend as the scaling factor multiplied. In contrast, task error, F_RMS_, XC_FTmax_, and PF_0.5Hz_ exhibited a decreasing trend with scaling factor increment.

**Table 1:** **The contrast of force variables among the low scaling factor (LSF), normal scaling factor (NSF), and high scaling factor (HSF) conditions**.

	**LSF**	**NSF**	**HSF**	**Statistics**
Task Error (% MVC)	0.951 ± 0.129^a^	0.627 ± 0.047^a^	0.671 ± 0.050^a^	***F***_**(2, 26)**_ **=** **8.78**, ***p*** **= 0.001**
F_RMS_ (% MVC)	0.991 ± 0.050^b^	0.828 ± 0.008	0.778 ± 0.0222^b^	***F***_**(2, 26)**_ **=** **10.64**, ***p*** **< 0.001**
F_SampEn_	0.172 ± 0.008^c^	0.192 ± 0.007^c^	0.197 ± 0.009^c^	***F***_**(2, 26)**_ **=** **25.44**, ***p*** **< 0.001**
XC_FTmax_	0.805 ± 0.019^d^	0.726 ± 0.036^d^	0.660 ± 0.041^d^	***F***_**(2, 26)**_ **=** **12.12**, ***p*** **< 0.001**
Lag Time (S)	−0.171 ± 0.008^e^	−0.049 ± 0.016^e^	0.037 ± 0.031^e^	***F***_**(2, 26)**_ **=** **12.44**, ***p*** **< 0.001**
Mean Frequency (Hz)	0.585 ± 0.009^f^	0.635 ± 0.017^f^	0.671 ± 0.020^f^	***F***_**(2, 26)**_ **=** **20.93**, ***p*** **< 0.001**
PF_0.5Hz_	0.158 ± 0.006^g^	0.140 ± 0.010	0.124 ± 0.009^g^	***F***_**(2, 26)**_ **=** **17.72**, ***p*** **< 0.001**

a *LSF > NSF, HSF, p ≤ 0.011*.

b *LSF > HSF, p = 0.002*.

c *LSF < NSF, HSF, p ≤ 0.002*.

d *LSF > HSF, NSF > HSF, p ≤ 0.008*.

e *LSF < NSF, HSF, p ≤ 0.003*.

f *LSF < NSF < HSF, p ≤ 0.003*.

g *LSF > HSF, p ≤ 0.001*.

*Bold values highlights the statistic results with significant difference*.

### Discharge patterns of motor units

**Figure 5:**
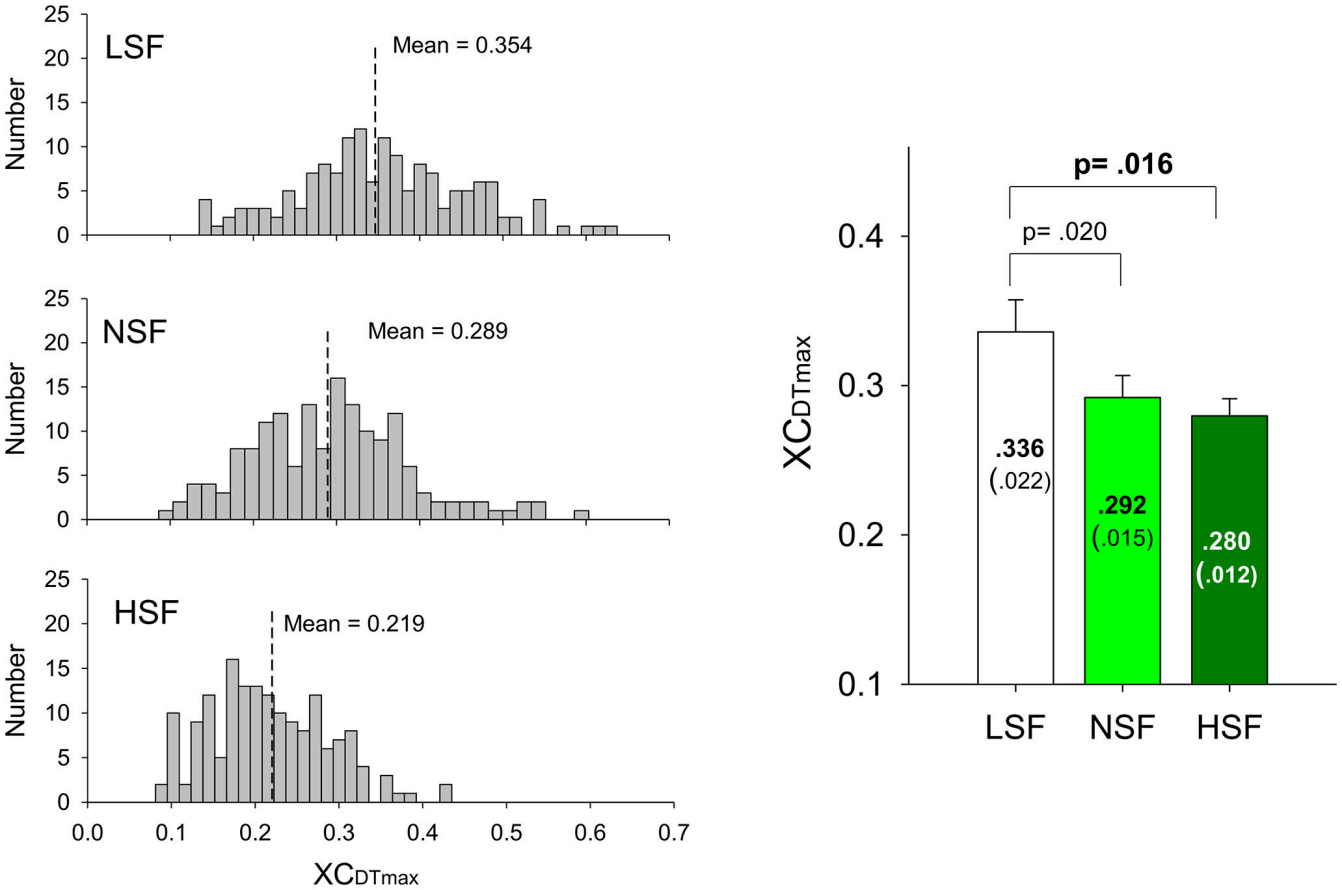
**Frequency distributions of peak cross-correlations of the motor unit discharges and target signals of a typical subject in the LSF, NSF, and HSF conditions (left plots)**. Means and standard errors of peak cross-correlations between the detrended discharge traces and target signals (XC_DTmax_) of all motor units for the different feedback conditions (right plot). (LSF, low scaling factor; NSF, normal scaling factor; HSF, high scaling factor).

**Figure 6:**
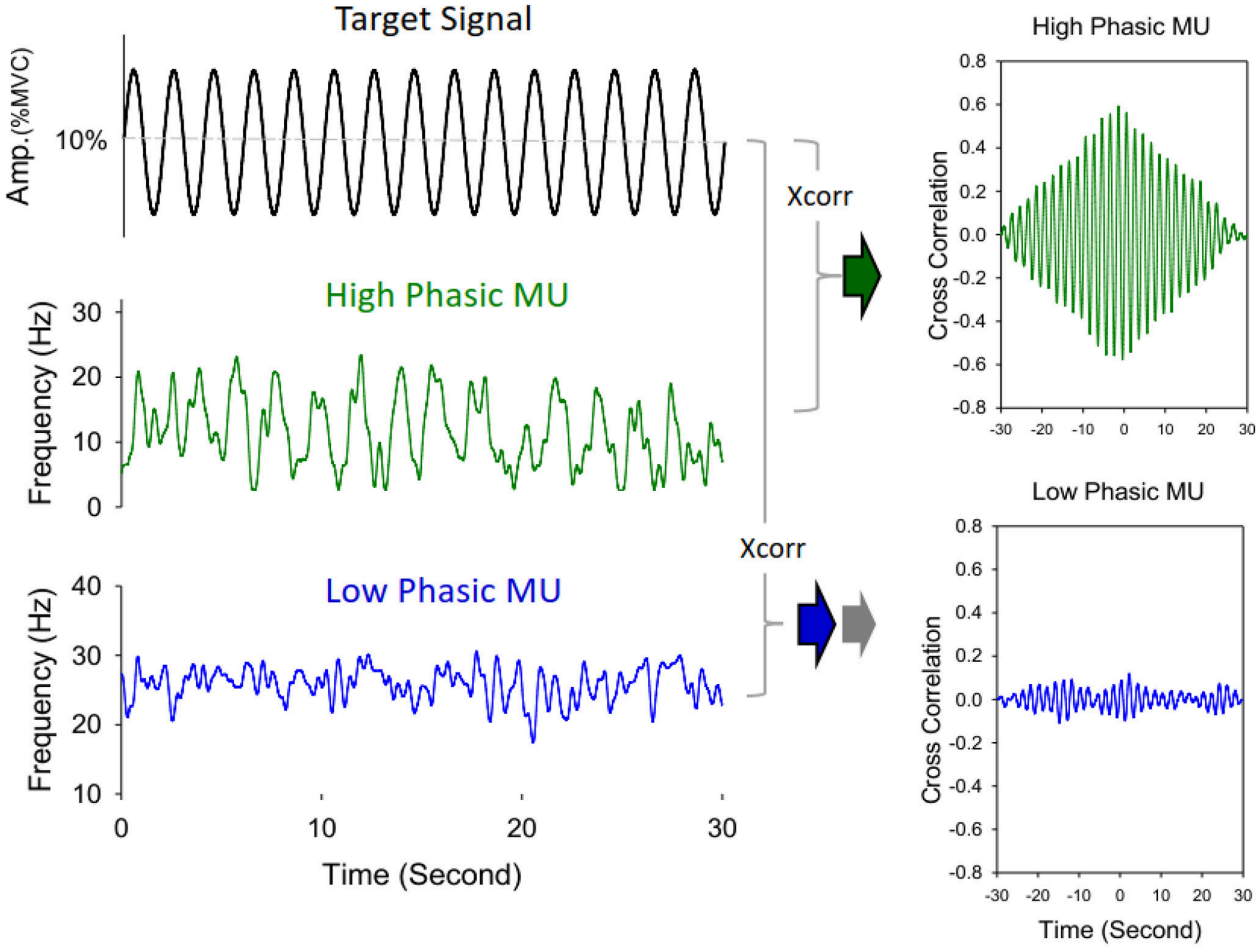
**Schematic illustration of identification of high phasic (HP) and low phasic (LP) motor units**. The discharge pattern of a high phasic motor unit exhibits a greater peak value of cross-correlation with the target signal, in contrast to a low phasic motor unit, which shows a lower correlation peak with the target signal.

The average numbers of decomposed (MUs) of an experimental trial and overall decomposition accuracy of the MUs did not vary with the feedback conditions (*p* > 0.05; Table 2). The left plot of Figure 5 shows the frequency distribution of the XC_DTmax_ of all MUs for the five experimental trials in each feedback condition from a typical participant. When the scaling factor increased, there was a downward shift of XC_DTmax_ for all identifiable MUs. The right plot of Figure 5 contrasts the population means of XC_DTmax_ among the three feedback conditions across the participants. The ANOVA test revealed that the scaling factor significantly affected the mean XC_DTmax_ [*F*_(2, 26)_ = 20.93, *p* < 0.001]. The LSF condition tended to exhibit a greater XC_DTmax_ than the NSF (*p* = 0.02) and HSF (*p* = 0.016) conditions did. Figure 6 shows two different types of MUs, (HP) and low phasic (LP) MUs, according to the degree of the cross-correlation of the instantaneous discharge rate and the target signal. The HP and LP MUs were denoted as those MUs with top 20% and bottom 80% of the peak cross-correlation of the discharge trace and the target signal (XC_DTmax_), respectively. Table 3 contrasts the mean values of ISI for the all, HP, and LP MUs in the LSF, NSF, and HSF conditions. Surprisingly, the ISIs of all the MUs did not vary with the scaling factor (*p* > 0.05). Hence, we further compared the mean ISIs of the HP and LP MUs among the various feedback conditions. Only the mean ISI of the HP MUs was a function of scaling factor (*p* < 0.05), rather than that of the LP MUs (*p* > 0.05).

**Table 2:** **The contrast of decomposition variables among the low scaling factor (LSF), normal scaling factor (NSF), and high scaling factor (HSF) conditions**.

	**LSF**	**NSF**	**HSF**	**Statistics**
Number of MU	31.2 ± 1.3	32.0 ± 1.9	32.3 ± 1.5	*F*_(2, 26)_ = 0.80, *p* = 0.459
Decomposition Accuracy (%)	95.3 ± 0.3	95.4 ± 0.3	95.4 ± 0.4	*F*_(2, 26)_ = 0.09, *p* = 0.917

**Table 3:** **The mean and standard errors of the mean inter-spike intervals (ISIs) of all, the high phasic (HP), and the low phasic (LP) motor units (MUs) for the different feedback conditions**.

**Unit: ms**	**LSF**	**NSF**	**HSF**	**Statistics**
All MU	55.6 ± 2.0	57.0 ± 2.0	57.0 ± 2.1	*F*_(2, 26)_ = 0.49, *p* = 0.620
HP MU	63.4 ± 3.3^a^	59.7 ± 3.4	58.8 ± 3.1^a^	***F***_**(2, 26)**_ **=** **4.24**, ***p*** **=** **0.026**
LP MU	54.1 ± 1.9	55.0 ± 2.0	54.4 ± 2.3	*F*_(2, 26)_ = 0.63, *p* = 0.539

LSF, low scaling factor; NSF, normal scaling factor; HSF, high scaling factor

a *LSF > HSF, p = 0.013*.

*Bold values highlights the statistic results with significant difference*.

### Central entrainment of the HP motor units

**Figure 7:**
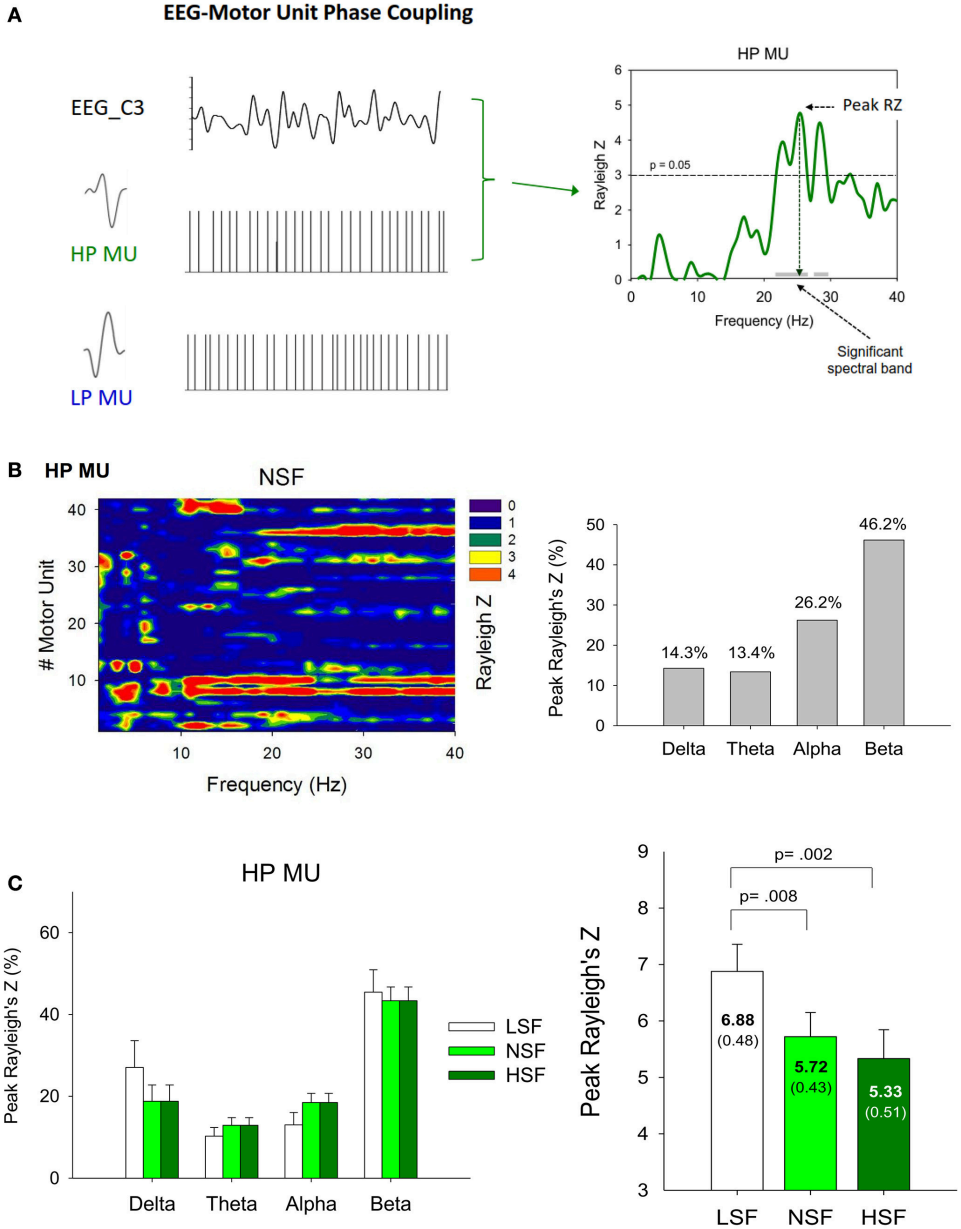
**(A)** Analysis of phase synchronization between a single high phasic (HP) motor unit (SMU) with oscillatory EEG activity of the C3 electrode (EEG C3). The left column is an example of an EEG and a single high phasic and low phasic motor unit recording. To quantify the cortical entrainment of the spike timing for the SMU, the Rayleigh z-statistic was calculated for each frequency component of the EEG signal and the timing of motor unit action potentials. The phase-locking profile of the representative HP motor unit is shown in the right column. **(B)** Summary of high phasic motor unit-EEG C3 phase locking for a typical participant in the normal scaling factor condition. The left plot summarizes the results of analyzing the HP motor unit-EEG C3 phase locking for all five experimental trials from a typical participant. The right plot represents the percentage of the spectral bands [delta (1–3 Hz), theta (4–7 Hz), alpha (8–12 Hz), and beta (13–35 Hz)] wherein the peak Rayleigh's Z of phasic motor units occurred. **(C)** The contrast of the population means of the percentages of the spectral bands wherein the peak Rayleigh's Z of high phasic motor units occurred and peak Rayleigh's Z among the different feedback conditions. (LSF, low scaling factor, NSF, normal scaling factor, HSF, high scaling factor).

Figure 7A shows the results of phase synchronization between a SMU of the HP MUs and EEG of the contralateral motor cortex at various spectral bands, with a significance level of Rayleigh's *Z* >3. The right plot of Figure 7A shows that the discharge of the representative HP MU was phase-locked to the EEG in the beta (13–35 Hz) band. The peak phase-locked activity took place at around 26 Hz. The left plot of Figure 7B displays the phase-locking profile of all the HP MUs of the five experimental trials from a representative participant in the NSF condition. The color of each pixel represents the degree of SMU phase synchronization (Rayleigh *z*-value) to EEG C3 at different spectral bands. Some MUs were phase-locked to a single spectral band of the EEG, while a portion of MUs could synchronize with EEG at more than two spectral bands. Certain MUs did not show a significant phase relationship with the EEG spectrum. The percentages of the spectral bands where the peak Rayleigh *z*-value occurred in this case is summarized in the right plot of Figure 7B. Figure 7C contrasts the population mean of the peak Rayleigh *z*-value (the right plot) and the percentages of the spectral bands for the peak Rayleigh *z*-value (the left plot). The ANOVA test revealed that the peak Rayleigh *z*-value of the SMU-EEG phase locking significantly varied with the scaling factor [*F*_(2, 26)_ = 7.23, *p* = 0.008]; the peak Rayleigh *z*-value was larger in the LSF condition than in the NSF and HSF conditions (*p* < 0.005). However, the percentage of each spectral band wherein the peak Rayleigh z-value occurred did not vary with the scaling factor [Delta (1–3 Hz): *F*_(2, 26)_ = 1.62, *p* = 0.217; Theta (4–7 Hz): *F*_(2, 26)_ = 0.582, *p* = 0.567; Alpha (8–12 Hz): *F*_(2, 26)_ = 1.75, *p* = 0.194; Beta (15–35 Hz): *F*_(2, 26)_ = 0.16, *p* = 0.850].

### Force-discharge relation

Table 4 summarizes the force-discharge relations of all, the (HP), and the (LP) MUs, in terms of peak cross-correlation of the force output and the cumulative spike trains of the MUs. The ANOVA statistics revealed that the peak cross-correlations of the force output and discharge traces of the HP MUs were significantly different among the feedback conditions (*p* < 0.05), and that the peak cross-correlation of the HP MUs in the LSF condition was significantly greater than that in the HSF condition (*p* < 0.0167). However, the peak cross-correlations for all MUs and the LP MUs did not significantly vary with the scaling factor (*p* > 0.05).

**Table 4:** **The force-discharge relation of all, the high phasic (HP), and the low phasic (LP) motor units in terms of peak cross-correlation**.

	**LSF**	**NSF**	**HSF**	**Statistics**
All MU	0.598 ± 0.032	0.541 ± 0.035	0.538 ± 0.031	*F*_(2, 26)_ = 2.99, *p* = 0.068
HP MU	0.637 ± 0.028^a^	0.569 ± 0.036	0.555 ± 0.027^a^	***F***_**(2, 26)**_ **=** **6.29**, ***p*** **= 0.006**
LP MU	0.534 ± 0.032	0.493 ± 0.029	0.505 ± 0.023	*F*_(2, 26)_ = 1.58, *p* = 0.224

LSF, low scaling factor; NSF, normal scaling factor; HSF, high scaling factor

a *LSF > HSF, p = 0.012*.

*Bold values highlights the statistic results with significant difference*.

## Discussion

### Behavioral mechanisms of force-tracking with variant error feedback sizes

Consistent with previous works (Emken and Reinkensmeyer, 2005; Patton et al., 2013; Abdollahi et al., 2014), this study also showed that the task quality of a force task was subject to the virtual size of the error feedback. The control of continuous cyclic force-tracking can be modeled as a continuum from feedback to feedforward (Pew, 1974; Miall et al., 1986; Huang et al., 2014; Inoue and Sakaguchi, 2014), in light of the phase lead or lag of the tracking response relative to the target signal. Slow feedback-based force-tracking exhibits more intermittencies than fast force-tracking predominated by the feedforward process, due to the more frequent corrective attempts based on greater utilization of spatial information from the visual feedback (Roitman et al., 2004; Pasalar et al., 2005; Selen et al., 2006). The behavior contexts of the change in virtual size of error feedback resembled those of rate-dependent force-tracking. Decreasing the virtual size of the error feedback increased the characteristics of fast force-tracking, including increases in phase leads and complexity reduction with mean frequency closer to that of the target in the LSF condition (Table 1). When the participants were misled by smaller virtual errors, they tended to make use of pattern-generation for cyclic force production due to the misrepresentation of target exactness. In contrast, the participants in the HSF condition became attentive to the visually-exaggerated mismatches, employing the evaluative function of the anterior cingulate cortex (Gehring and Fencsik, 2001; Rodriguez-Fornells et al., 2002). The enhanced response conflict suppressed the force control with pattern generation, replacing it with the feedback process to correct error at a shorter time scale. This paradigm shift reduced the PF_0.5Hz_ but increased the high-frequency force intermittency (Figure 4). The greater force complexity and increasing lag time further supported the increasing use of visual information to optimize the force task with a richer error-correction strategy due to the virtual enhancement of errors (Liu and Todorov, 2007; Chen et al., 2013; Lin et al., 2014).

### Adaptation of motor unit behaviors to varying sizes of error feedback

The above-mentioned modulation of force behaviors is supported by neurophysiological evidences in the form of the coherent MU discharge (Figure 5) and force-discharge relation (Table 4). The degree of coherent MU discharge to the target signal increased when the virtual size of the error feedback was smaller, and higher force-discharge relation was noted in the LSF condition. The size-related changes in the coherent discharge of MUs bore a striking resemblance to the rate-dependent variations in MU discharge (Sosnoff et al., 2005). In a force-tracking task, a faster target rate was associated with more narrowband EMG power spectra at the target frequency, because the feedforward process could expedite faster oscillatory contractions with enhanced coherent MU discharge to the target movement (Sosnoff et al., 2005; De Luca et al., 2014). The degree of coherent MU discharge was tuned to the virtual size of the error feedback in this study. Owing to the small size of the perceived error, the participants in the LSF condition presumed an optimal expected state for force-tracking that encouraged emphasis on the more effective feedforward mechanism to track the target movement with a stronger coherent MU discharge. In contrast, the brain weakened the coherent MU discharge when central pattern generation was used, and this approach was not effective for proper adaptation to the overt response mismatches in the HSF condition.

Surprisingly, the mean ISI for all MUs was not contingent on the virtual size of the error feedback (Table 3). Therefore, we classified the MUs as (HP) and (LP) MUs, according to the degree of the target constraints on MU discharge (Maton, 1976; Iyer et al., 1994). Conceptually, the distinctive discharges of the HP and LP MUs support the use of the MU task group for erroneous responses. The idea of the MU task group posits that different subsets of MUs in a muscle could flexibly discharge to meet various task needs (such as isometric vs. dynamic Enoka et al., 1989; Jones et al., 1994; Mohr et al., 2015; isometric vs. isotonic Thomas et al., 1986). Apart from the central influence that could be exerted on the after-hyper-polarization trajectory during repetitive MU discharges, the intrinsic properties of the motoneurons are modifiable to specific task needs (Sturm et al., 1997). In addition, the synaptic noise (Matthews, 1996) and independent noise that are input to modify MU discharge could be task dependent (Farina and Negro, 2015; Farina et al., 2016). Although the exact cause of the MU task group in this study is not fully clear, only the HP MUs that transformed the neural input to cyclic mechanical output were tuned to the size of the erroneous response, whereas the LP MUs seemed to play a compensatory role in force maintenance around a fixed level (Riley et al., 2008).

### Central mechanism effects on the motor units susceptible to error feedback

Another novel finding of this study was that the peak value of the SMU-EEG phase coupling of the HP MUs increased with error-reducing feedback (Figure 7C). This fact clearly suggested that the discharge of the HP MUs was centrally modulated, and that it was subservient to augmentation of the coherent discharge of the HP MUs to the target signal in the LSF condition. The error-related cortical potential in the motor area has not attracted much attention, except a report by Anguera et al. (2009) that the time course of movement potentials recorded in the primary motor cortex was greater in large error trials than in small error trials during a sensorimotor adaptation task. Being time-locked to the onset of a corrective submovement, the movement potentials were thought to initiate a corrective response and its completion. However, unlike the frontal midline theta activity, which was time-locked to the initiative visual cue to monitor action outcomes (Luu et al., 2004; Arrighi et al., 2016), the spectral ranges of the peak SMU-EEG phase coupling of the HP MUs did not vary with the virtual size of the error feedback (Figure 7C). Hence, the synchronized discharge of the HP MUs could not be ascribed to specific cortical oscillatory activity in the primary motor cortex, which defines the brain state of remedial action.

### Methodological issues

With state-of-the-art decomposition techniques, the discharge patterns of MUs were characterized from multi-electrode surface EMG, even though the selectivity of this approach could be poorer than that of intramuscular EMG for the superposition of motor unit action potentials. To be rigorous, we conducted the experiment at a low exertion level (around 10% MVC). For the human first dorsal interosseous muscle at low levels of muscle contraction, the high overall validity (approximately 95%) of the present surface EMG decomposition algorithm was confirmed by comparing the spike timings of simultaneous recordings of intramuscular and surface EMGs (Hu et al., 2014). We also applied the “reconstruct-and-test” procedure to support the accuracy of the obtained identifications (De Luca et al., 2015a). In practical reality, the decomposition accuracy was satisfactorily high (91.50–96.65%), supporting the possibility of MU detection in the FDI muscle (De Luca et al., 2006; Nawab et al., 2010). Although the accuracy of surface EMG decomposition is still debatable (Farina and Enoka, 2011; De Luca et al., 2015a), it is beyond the scope of the present setup to resolve pre-existing disputes over surface EMG decomposition. In the least, the motor unit behaviors during cyclic contraction observed in this study were largely consistent with those of previous reports (Iyer et al., 1994; Knight and Kamen, 2007; Erimaki et al., 2013). On the other hand, one technical merit of using surface EMG decomposition is that it can detect more active MUs than intramuscular EMG can, which helps to characterize error-dependent discharges in a small portion of the HP MUs. However, we cannot deny the possibility that even a small decomposition error of surface EMG signals could bias estimation of the correlation between force fluctuations and discharge of phasic MUs (Piotrkiewicz and Türker, 2017), particularly for classification of the LP MUs. Therefore, this study reported that only the HP MUs were tuned to the feedback error, whereas the discharge characteristics of the LP MUs and the common drive of all MUs were independent of the scaling factor (cumulative pooled MU coherence (Farina and Negro, 2015; Farina et al., 2016) is not shown for brevity).

In addition, this study did not account for atypical force regulation under the conditions of extreme scaling factors. We agree that exceptional cases should be reinvestigated in future studies. For instance, the use of very large scaling factors could degrade cyclic force-tracking because excessively large feedback gain could destabilize closed-loop behaviors in the human visuomotor system (Navas and Stark, 1968). It is necessary to emphasize that manipulation of an error scaling factor during force-tracking affects the functional integrities of the performance monitoring system differently than do changes in visual gain (or pixel/force ratio) (Lee Hong and Newell, 2008; Kuznetsov and Riley, 2010; Kennedy and Christou, 2011; Jordan et al., 2013). At a fixed pixel/force ratio, the use of force-tracking under the different scaling factor conditions in this study was intended to alter the frontal attentive control over mismatch during the force task. This approach was chosen so that the virtual size of the execution error would alter the representation of the predicted “correct response” during the force-tracking and thereby activate the executive function for error monitoring and correction (Gehring and Fencsik, 2001; Rodriguez-Fornells et al., 2002). In contrast, visual gain would determine the spatial quality of both the force output and target signals on the display. The behavior effects corresponded to visual gain alter visuomotor processing and the degrees of somatotopic organization, involving in bilateral premotor area and right inferior parietal lobule (Coombes et al., 2010, 2011; Prodoehl and Vaillancourt, 2010). Therefore, variations in spatiotemporal tuning of brain activity and force performance with respect to visual gain and error scaling factor are different. Under the condition of continuous visual feedback, no previous studies have proved that variations in force control due to changes in visual gain can be ascribed to a paradigm shift along a continuum from feedback to feedforward processes.

## Conclusions

This study is the first to shed light on the behavioral and neurophysiological mechanisms of variations in force control with the virtual size of execution error. Error-reducing feedback favors a feedforward process, and force-scaling manifests with fewer corrective attempts. Error-reducing feedback also adds to force-discharge relation due to a significant increase in the coherent discharge of (HP) MUs with the target rate. In contrast, force control with error-enhancing feedback is subject to a feedback process, which occurs concurrently with increases in high-frequency force components and force complexity. The reliance on the feedback process suppresses the coherent discharge of the (HP) MUs with the target rate and decouples the force-discharge relation. Manipulation of the virtual size of error feedback leads to a paradigm shift in force control due to underlying selective central modulation of the discharge pattern of the HP MUs.

## Author contributions

Substantial contributions to the conception or design of the work; or the acquisition, analysis, or interpretation of data for the work conception or design of the work, IH andYC. Acquisition, YC and IH. Analysis, IH, GC, and YC. Interpretation of data, YC, IH, and YL. Drafting the work or revising it critically for important intellectual content, YC, IH, and YL. Final approval of the version to be published: IH and YC. Agreement to be accountable for all aspects of the work in ensuring that questions related to the accuracy or integrity of any part of the work are appropriately investigated and resolved: YC, IH, and YL.

## Funding

This research was supported by grants from the Ministry of Science and Technology, Taiwan, R.O.C., under Grant No. 103-2410-H-040-010-MY2 and 104-2314-B-006-016-MY3.

### Conflict of interest statement

The authors declare that the research was conducted in the absence of any commercial or financial relationships that could be construed as a potential conflict of interest.

## Abbreviations

EEG: electroencephalography
EMG: electromyography
FDI: first dorsal interosseous
F_RMS_: root mean square value of detrended force output
F_SampEn_: Sample entropy of detrended force output
HP: High phasic
HSF: High scaling factor
ISI: inter-spike interval
LP: Low phasic
LSF: Low scaling factor
MF: mean frequency
MU: motor unit
MUAP: motor unit action potential
MUAPT: motor unit action potential train
MVC: maximal voluntary contraction
NSF: Normal scaling factor
RE: real error
RF: real force
PF_0.5Hz_: normalized amplitude of peak frequency at 0.5 Hz
RMS: root mean square
SampEn: sample entropy
SMU: single motor unit
T: target signal
VE: visualized error
VF: visualized force
XC_DTmax_: peak cross-correlation of the detrended discharge rate and target signal
XC_FTmax_: peak cross-correlation of the detrended force output and target signal.
